# Keeping in touch with the visual system: spatial alignment and multisensory integration of visual-somatosensory inputs

**DOI:** 10.3389/fpsyg.2015.01068

**Published:** 2015-08-05

**Authors:** Jeannette R. Mahoney, Sophie Molholm, John S. Butler, Pejman Sehatpour, Manuel Gomez-Ramirez, Walter Ritter, John J. Foxe

**Affiliations:** ^1^The Cognitive Neurophysiology Laboratory, The Nathan S. Kline Institute for Psychiatric Research, OrangeburgNY, USA; ^2^Division of Cognitive and Motor Aging, Department of Neurology, Albert Einstein College of Medicine, New YorkNY, USA; ^3^The Sheryl and Daniel R. Tishman Cognitive Neurophysiology Laboratory, Children’s Evaluation and Rehabilitation Center, Department of Pediatrics, Albert Einstein College of Medicine and Montefiore Medical Center, New YorkNY, USA; ^4^The Dominick P. Purpura Department of Neuroscience, Rose F. Kennedy Center, Albert Einstein College of Medicine, New YorkNY, USA

**Keywords:** visual-somatosensory integration, multisensory integration, cross-modal, sensory processing, high-density electrical mapping

## Abstract

Correlated sensory inputs coursing along the individual sensory processing hierarchies arrive at multisensory convergence zones in cortex where inputs are processed in an integrative manner. The exact hierarchical level of multisensory convergence zones and the timing of their inputs are still under debate, although increasingly, evidence points to multisensory integration (MSI) at very early sensory processing levels. While MSI is said to be governed by stimulus properties including space, time, and magnitude, violations of these rules have been documented. The objective of the current study was to determine, both psychophysically and electrophysiologically, whether differential visual-somatosensory (VS) integration patterns exist for stimuli presented to the same versus opposite hemifields. Using high-density electrical mapping and complementary psychophysical data, we examined multisensory integrative processing for combinations of visual and somatosensory inputs presented to both left and right spatial locations. We assessed how early during sensory processing VS interactions were seen in the event-related potential and whether spatial alignment of the visual and somatosensory elements resulted in differential integration effects. Reaction times to all VS pairings were significantly faster than those to the unisensory conditions, regardless of spatial alignment, pointing to engagement of integrative multisensory processing in all conditions. In support, electrophysiological results revealed significant differences between multisensory simultaneous VS and summed V + S responses, regardless of the spatial alignment of the constituent inputs. Nonetheless, multisensory effects were earlier in the aligned conditions, and were found to be particularly robust in the case of right-sided inputs (beginning at just 55 ms). In contrast to previous work on audio-visual and audio-somatosensory inputs, the current work suggests a degree of spatial specificity to the earliest detectable multisensory integrative effects in response to VS pairings.

## Introduction

Human ERP studies have shown that when information from various sensory modalities is presented concurrently, multisensory interactions often occur within the first 100 ms post-stimulation (e.g., Schroger and Widmann, 1998; Giard and Peronnet, 1999; Foxe et al., 2000; Fort et al., 2002; Molholm et al., 2002, 2004; Schürmann et al., 2002; Foxe and Schroeder, 2005; Murray et al., 2005). For example, Giard and Peronnet (1999) detailed a series of ERP modulations attributable to auditory-visual (AV) integration where the earliest multisensory interaction was found to begin at just 40 ms over visual cortex. This finding was questioned justifiably by Teder-Sälejärvi et al. (2002) on the grounds that a potential confound was introduced by the analysis method employed due to the presence of anticipatory potentials during each unisensory event ^1^. However, comparably early AV integrations were reported by Molholm et al. (2002) when such factors were properly controlled for by randomly varying the inter-stimulus intervals over a wide range. Similarly, Foxe et al. (2000) showed auditory-somatosensory (AS) interactions at just 50 ms, a finding corroborated and extended by Gonzalez Andino et al. (2005) and Murray et al. (2005).

In all of these studies, in addition to the consistent finding of early multisensory interactions, spatio-temporal mapping has also revealed a family of subsequent multisensory processing stages across a widely distributed network of sensory and higher-order regions. While considerable strides have been made in detailing the regions and time frames of MSI for the various sensory combinations, very little is yet known about the specific functional consequences of a given multisensory effect. Much of the current work has been guided by the seminal work of Stein, Meredith, Wallace and colleagues who, in a series of studies using single-unit recordings in the superior colliculus (SC) of cats and monkeys, detailed a basic set of principles for MSI (Stein et al., 1975, 1993; Meredith and Stein, 1986; Meredith et al., 1987; Stein and Wallace, 1996; Wallace et al., 1996). They showed that integration in SC neurons was greatest for inputs presented simultaneously or in close temporal coincidence (the temporal principle), that the magnitude of the multisensory effect was inversely related to the effectiveness of the constituent unisensory inputs (the inverse-effectiveness principle), and of particular importance to the current study, that MSI was greatest for stimuli presented to the same spatial location (the spatial rule). In the ongoing attempt by ERP and neuroimaging researchers to detail the functional significance of the aforementioned cortical integration effects, these principles have provided a solid launching point (see Foxe, 2008).

In an effort to determine whether spatial alignment was a critical parameter for early multisensory AS interactions, Murray et al. (2005) presented spatially aligned and misaligned AS multisensory combinations to both the left and right hemifields. In the misaligned conditions, the constituent auditory and somatosensory elements were presented over 100 degrees apart; a distance that left no spatial ambiguity regarding the separation of the two inputs. Results revealed that the earliest AS multisensory interactions detectable in cortex (at just 50–95 ms) were not constrained by spatial alignment. It has since become clear that these early integration effects have significant impact on behavior in terms of speeded responses to multisensory inputs (see Sperdin et al., 2009). Similarly, Fiebelkorn et al. (2011) demonstrated behavioral AV integration effects where auditory inputs facilitated visual target detection regardless of retinal eccentricity and large misalignments of the audiovisual stimulus pairings. Teder-Sälejärvi et al. (2005) also examined the effect of spatial alignment on multisensory AV interactions. Consistent with the findings of Murray et al. (2005), their results indicated clear facilitation of RTs to multisensory AV conditions regardless of spatial alignment. Using saccadic reaction times (RTs) as endpoints, work using the sensory pairing of current interest [i.e., visual-somatosensory (VS) inputs], showed clear speeding of responses to visual targets when they were paired with a tactile input, and this was the case when tactile inputs were as much as 110° apart from the visual input (Diederich et al., 2003). However, there did appear to be a modicum of spatial specificity in this study in that saccades were faster again when the tactile inputs were ipsilateral to the visual target rather than contralateral, so the picture is somewhat unclear as to the specificity of such spatial effects (see also Diederich and Colonius, 2007).

To our knowledge, only one study has examined early VS multisensory ERP interactions (Schürmann et al., 2002), and the express purpose of that study was to assess issues of spatial alignment. Participants passively observed visual and somatosensory stimuli, which were presented in a blocked design, wherein only a single stimulus type was presented at a time. Somatosensory stimuli were only presented to the left wrist using median nerve electrical stimulation, whereas visual stimuli were presented to both hemifields from a computer screen that was placed fully 1.5 m in front of the participants. Although it was not specified exactly where the arm was placed or how far apart the left and right visual stimuli were^2^, the physical setup did not afford spatial coincidence of somatosensory and visual stimuli. A more thorough examination using randomly presented somatosensory, visual and multisensory VS stimulation to both left and right hemifields, high-density mapping and complementary psychophysical data is clearly warranted.

The purpose of the current study was to assess whether spatial alignment is critical for early VS interactions in young adults. There is good basis for thinking that VS processing should be more spatially constrained than AS or AV processing. In terms of spatial acuity, the auditory system is quite susceptible to spatial capture by both the somatosensory (e.g., Soto-Faraco et al., 2004) and visual systems (i.e., Ventriloquism: Bertelson, 1999; Soto-Faraco et al., 2002). In Murray et al. (2005), we found that the earliest AS interactions were localized to auditory cortical regions, and yet the laterality of this effect was tied to the side of somatosensory stimulation rather than auditory stimulation; suggesting that the more precise spatial information available to the somatosensory system dominated during early sensory-cortical integration. These findings are consistent with the so-called “modality-appropriateness” hypothesis, which posits that the modality with the highest processing resolution for a given feature will dominate integrative cross-sensory processing of that feature (Welch and Warren, 1980; Shimojo and Shams, 2001). But, what of integration across two senses that both have high resolution for a given feature?

Given the high spatial resolution of both the visual and somatosensory systems, it seems reasonable to propose that spatially misaligned inputs will simply not be integrated during early processing. In support, Pavani et al. (2000) have provided psychophysical evidence for greater RT facilitation to aligned visual-tactile stimuli. Using a paradigm that required participants to place their hands under a table and fixate a light on top of a table, Pavani et al. (2000) revealed robust congruency effects for aligned versus misaligned tactile vibrations and task-irrelevant, non-informative light flashes presented over the hand locations. In a very clever manipulation, they showed an amplification of this alignment effect when an additional set of “fake” hands (a pair of stuffed rubber gloves) were arranged in front of the subjects such that they were directly above and aligned with the exact placement of the participant’s hands beneath the table. Although the hands were not their own, the participants reported a strong sense that the fake hands were indeed their own and the congruency effect for tactile inputs and the non-informative light stimuli was increased. However, this effect collapsed when the rubber hands were reoriented (90° out of alignment) with regards to the position of the participant’s hands, effectively obliterating the illusion that the hands could be the participant’s own. Data such as these suggest that cortical multisensory processing should be highly sensitive to spatial alignment across vision and touch, but they do not address at which stage of processing this spatial sensitivity emerges. Here, we set out to determine whether the earliest stages of VS integrative processing in cortex would be similarly insensitive to spatial alignment as previously demonstrated for AV and AS combinations, or whether the high spatial resolution of both the visual and somatosensory systems would lead to a different processing mode whereby spatial alignment plays a much more prominent role during integrative processing.

## Materials and Methods

### Participants

Fourteen (seven female), neurologically normal volunteers (mean age 26.07, ±4.41) participated in the current experiment. Data from an additional two participants were excluded: one because of equipment malfunction midway through the recording session and the second because of excessive EMG activity. All participants were right-handed as assessed by the Edinburgh handedness inventory (Oldfield, 1971) and had normal or corrected-to-normal vision. All participants provided written informed consent to the experimental procedures in accordance with the tenets of the Declaration of Helsinki and the Institutional Review Board of the Nathan Kline Research Institute approved all procedures. Participants received a modest monetary compensation for their service.

### Stimuli

Visual and somatosensory stimuli were produced from a custom built stimulus generator (Enabling Devices Inc., Hawthorne, NY, USA) that consisted of two 8 mm diameter red light emitting diodes (LEDs) with a luminosity intensity of 1600 mcd mounted on the left and right thumbs with Velcro and two 4 mm vibrator motors with 1G vibration amplitude attached to the left and right index fingers with 3 M Micropore tape. The stimuli were cycled on and off at precise intervals either alone or in combination through the computers parallel port. Participants wore mittens to ensure that the vibro-tactile stimulators were not visible. To ensure that the somatosensory stimuli were inaudible, each participant wore earplugs in combination with headphones over which continuous white noise was played. White noise levels were set at 60 dB SPL and were modified on an individual basis to ensure full-masking of any vibro-tactile stimulator sound.

**FIGURE 1:**
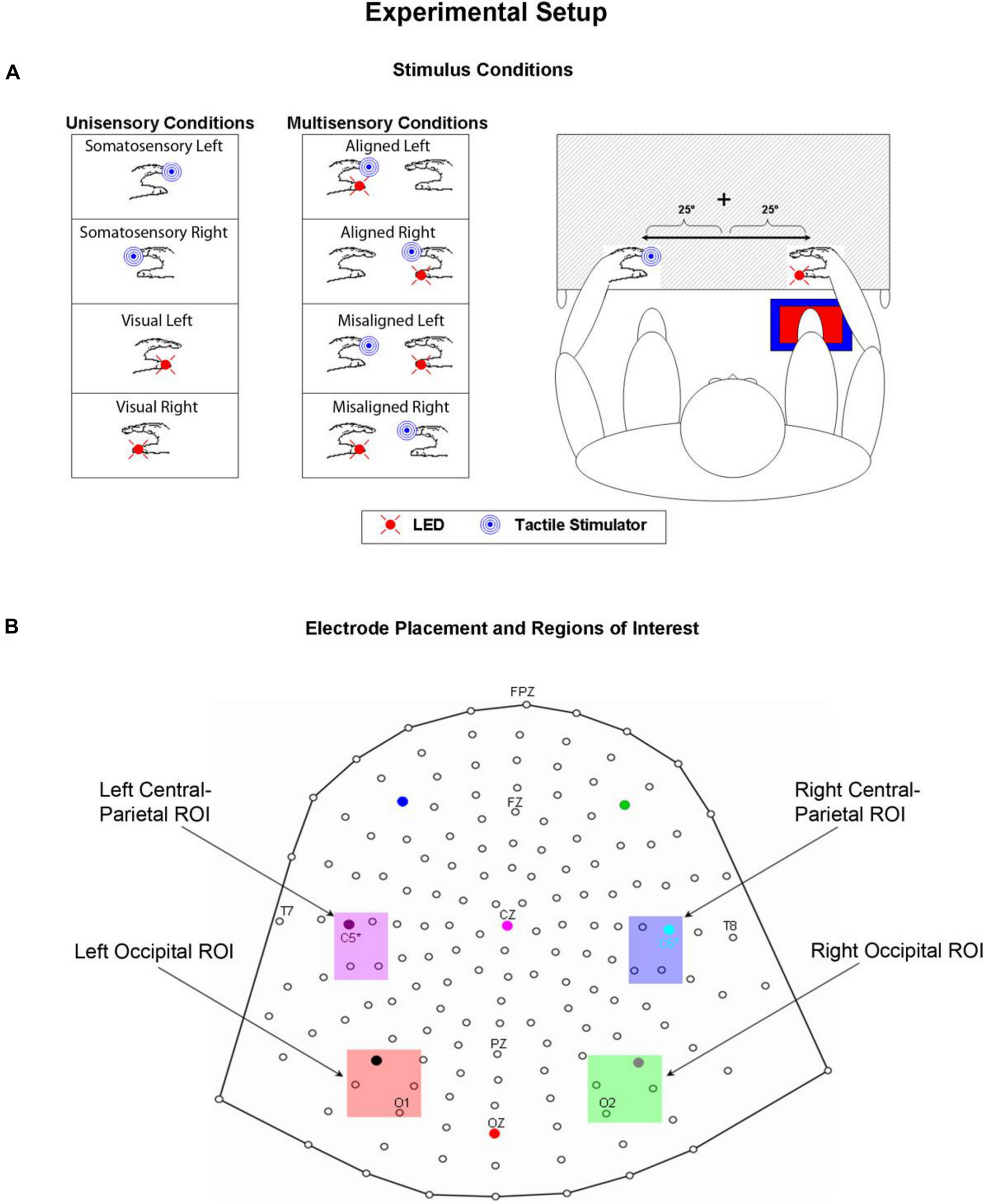
**Experimental Paradigm. (A)** Unisensory visual, unisensory somatosensory, and multisensory VS conditions. LEDs were placed on both left and right thumbs and vibro-tactile stimulators were placed on both left and right index fingers. Mittens were used to hide vibro-tactile stimulators and headphones were used to mask the sound of the vibrators. Participants sat comfortably with their hands rested on a table and used a foot pedal located under their right foot to respond to any and all stimuli. Stimuli were 25° from the azimuth. **(B)** 168 electrode array and the four selected regions of interest (ROIs) used for statistical analyses.

A TTL (transistor-transistor-logic, 5 volts, duration 60 ms) pulse was used to trigger the various stimuli through Presentation software. A total of eight stimulus conditions (four unisensory and four multisensory) were presented to the participants (see **Figure 1A**). The unisensory conditions included visual (V) and somatosensory (S) stimulation delivered to either the left or right hand. The multisensory conditions included spatially aligned simultaneous VS stimulation presented at the same location (e.g., left thumb and left index finger) and spatially misaligned simultaneous VS stimulation presented at different locations (e.g., left thumb and right index finger). The eight stimulus conditions were presented in random order with equal frequency in blocks of 200 trials. The average number of blocks that each participant completed was 24, permitting ∼600 trials of each of the eight stimulus conditions.

In terms of the time course of stimulus events, each trial commenced with a random inter-trial-interval (ITI) between 1 and 3 s. Next, one of the eight stimulus conditions was presented for 60 ms and the participant was given up to 2.5 s to respond. After the response was made, the next trial commenced with another random ITI. Note that the wide-ranging distribution of stimulus presentation timing protects against anticipatory effects (see Molholm et al., 2002).

### Task

Psychophysical and electrophysiological measures were collected as participants performed a simple RT task in response to somatosensory and visual stimuli by depressing a foot pedal located under their right foot for each and every stimulus event. Participants were asked to respond as quickly as possible to each stimulus (regardless of spatial location) whether it was seen, felt or both. Participants’ arms were rested on a table and their hands were exactly 60 cm apart, symmetrical about the vertical meridian. When aligned, the visual and somatosensory stimuli were separated by no more than 2.5 cm since they were attached to two different fingers on the same hand. Participants were required to fixate a central fixation point (a white cross) visible on the table surface throughout the entire experiment. The lateralized stimuli were presented 25° from the central fixation point; it was felt that any separation greater than 25° would have resulted in a weak visual evoked potential (VEP), not only because of the distance in the periphery, but also because of the small size of the LEDs. Participants were encouraged to take as many breaks as necessary between blocks to reduce fatigue and facilitate the maintenance of concentration.

### Behavioral Analysis

Behavioral data allow for a direct measurement of multisensory integrative processes through RTs. That is, when two sources of information (i.e., a light and a vibro-tactile stimulus) are presented at the same time, they offer redundant signals that give rise to faster detection responses, a phenomenon referred to as the RSE (Kinchla, 1974). Two very distinct models can be implemented to explain the RSE: race models and coactivation models (Miller, 1982). In race models, when two information sources are presented concurrently (e.g., a multisensory stimulus), the signal from the information source that is processed fastest is the signal that produces the response (i.e., the “winner” of the race). However, co-activation models are supported when RTs to multisensory stimuli are faster than would be predicted by race models. In the latter case, the RT facilitation is accounted for by interactions that allow signals from redundant information sources to integrate or combine non-linearly. For instance, Harrington and Peck (1998) successfully revealed facilitation of saccadic latencies that exceeded latencies predicted by the race model for multisensory AV stimuli that were separated by visual angles up to 17.5°.

For each participant, individual RTs to each stimulus were recorded. RTs were then sorted by stimulus condition and averaged. Trials with RT responses that exceeded ±2 SD from the individual mean of each participant were excluded (see also Brandwein et al., 2011; Mahoney et al., 2011); the percentage of discarded trials ranged from 4 to 6 percent loss per participant, across all stimulus conditions. The RT range within the valid RTs was calculated across the eight stimulus conditions and quantized into twenty bins from the first to the hundredth percentile in 5% increments (1, 5,…, 95, 100%).

Planned comparisons between each of the unisensory stimulus conditions and the simultaneous multisensory stimulus conditions were performed to test for a RSE (see **Table 1**). Upon evidence of a RSE, Miller’s (1982) inequality was used to establish whether there was a race model violation. The model places an upper limit on the cumulative probability (CP) of RT at a given latency for stimulus pairs. For any latency, *t*, the race model holds when this CP value is less than or equal to the sum of the CP from each of the unisensory stimuli minus an expression of their joint probability [CP_(t)simultaneous_ < ((CP_(t)unisensory1_ + CP_(t)unisensory2_) – (CP_(t)unisensory1_ × CP_(t)unisensory2_)); see also Molholm et al., 2002].

**Table 1:** Visual-somatosensory (VS) reaction time (RT) facilitation^a^.

Stimulus condition	RTs to simultaneous multisensory stimulus pair	RTs to VS constituent unisensory stimulus conditions		*T* value _(d.f.)_; *p*-value
Aligned Left	313 ms	Soma (left):	327 ms	*t*_(13)_ = 6.09; *p* < 0.001
		Visual (left):	369 ms	*t*_(13)_ = 9.69; *p* < 0.001
Aligned Right	312 ms	Soma (right):	329 ms	*t*_(13)_ = 6.29; *p* < 0.001
		Visual (right):	369 ms	*t*_(13)_ = 18.99; *p* < 0.001
Misaligned Left	312 ms	Soma (left):	327 ms	*t*_(13)_ = 5.25; *p* < 0.001
		Visual (right):	369 ms	*t*_(13)_ = 13.22; *p* < 0.001
Misaligned Right	314 ms	Soma (right):	329 ms	t_(13)_ = 5.77; *p* < 0.001
		Visual (left):	369 ms	t_(13)_ = 10.37; *p* < 0.001

*^a^Results from follow-up planned comparisons (paired *t*-tests) confirm that RTs to VS multisensory pairs (aligned and misaligned) were significantly shorter than any unisensory stimulus condition.*

### EEG Acquisition

High-density continuous electroencephalographic (EEG) recordings were acquired through the Active Two BioSemi electrode system from 168 scalp channels, digitized at 512 Hz. With the BioSemi system, any electrode can be assigned as the reference, which is done purely in software after acquisition. BioSemi replaces the ground electrodes that are used in conventional systems with two separate electrodes: Common Mode Sense (CMS) active electrode and Driven Right Leg (DRL) passive electrode. These two electrodes form a feedback loop, rendering them references. For a detailed description of the referencing and grounding conventions used by the Active Two BioSemi electrode system, visit www.biosemi.com/faq/cms&drl.htm.

Trials were epoched from 100 ms pre-stimulus to 500 ms post-stimulus and baseline was then defined over the -100 to 0 ms epoch. An artifact rejection criterion of ±100 μV was used to exclude trials with excessive EMG and other noise transients. The average acceptance rate of trials per condition was ∼73 ± 18.3% (with the minimum number of accepted sweeps equal to 415). Data from individual channels (scalp sites) that were noisy or faulty were interpolated based on data from neighboring electrode sites at the individual subject level.

Averages were generated based on four unisensory conditions: (1) V Left, (2) V Right, (3) S Left, (4) S Right, and four multisensory VS conditions: (1) Aligned Left, (2) Aligned Right, (3) Misaligned Left and (4) Misaligned Right. For identification purposes, the direction of the misaligned conditions, (e.g., “Misaligned Left”) referred to the position of the somatosensory stimulator, with concurrent visual stimulation always presented to the opposite hemifield. All averages were then re-referenced to a frontal-polar site (approximately FPz in the 10–20 EEG convention).

### EEG Analysis

To test for multisensory interactions between V and S inputs, responses to each of the multisensory stimulus conditions were compared to the summed responses of the constituent unisensory stimulus parts (i.e., “summed”). If the ERPs from the summed responses were equivalent to ERPs from the simultaneous responses, then one could argue that these two sets of neural responses were indeed independent and linear processes. However, any reliable difference between the summed and the simultaneous ERPs was indicative of non-linear interactions of the neural responses to the multisensory stimuli. It should be noted that this methodology will not be sensitive to areas of purely multisensory convergence wherein responses to two sensory modalities might occur, but would sum linearly (Foxe et al., 2002).

#### VS Interaction Analysis Strategy

In an effort to test for statistical differences between the ERPs of the multisensory VS conditions and the ERPs of the constituent unisensory V + S conditions, 20 ms time windows around the somatosensory P60 and N140 components over central scalp sites were selected. The same procedure was implemented for time windows around the earliest detectable visual activity (i.e., the C1 component) and the subsequent P1 and N1 VEP components over occipital scalp sites. The two symmetrical regions of interest (ROI) were chosen based on known topographies of these classical somatosensory and visual ERPS and consisted of a total of four electrodes (see **Figure 1B** for electrode placement and specific ROI locations). The center of each time window was indicative of the peak of each component in the mean waveform for the relevant unisensory condition, with the outer boundaries of the time window equal to ±10 ms from the peak. In addition to these time windows centered on specific visual and somatosensory components, 20 ms time window (110–130 ms) over left and right parieto-central scalp was selected for testing. Visual inspection of the group average data strongly suggested differences in simultaneous and summed activation during this time window that appeared to be indicative of integrative multisensory processing; thus, statistical analyses during this time window were deemed necessary.

Four-way repeated-measures ANOVAs (alpha criterion of 0.05) with factors of condition (multisensory simultaneous or summed unisensory), alignment (aligned or misaligned), stimulus presentation (left or right hemifield), and ROI (left or right hemi-scalp) were implemented for each of the six time windows of interest. Statistical significance was assessed with an alpha level of 0.05 and Greenhouse–Geisser corrections were used when appropriate.

A second *post hoc* exploratory stage of analysis was also undertaken using the so-called statistical cluster plot (SCP) method (see e.g., Guthrie and Buchwald, 1991; Molholm et al., 2002). Under this method, running dependent samples t-tests were performed between summed and multisensory conditions (for aligned and misaligned conditions) across all channels and time points. A clustering approach method was employed to control for inflation of Type I error due to multiple comparisons (cf., Guthrie and Buchwald, 1991). The rationale for this method is that Type I errors are unlikely to endure for several consecutive time points. However, since the EEG signal does not change arbitrarily fast, there is some dependence between consecutive time points, so correction for autocorrelation in the signal must be made. Based on Guthrie and Buchwald (1991), we thus required two-tailed *p*-values below 0.05 to persist for at least 10 samples (∼20 ms) to consider the effects significant. This approach gives an assessment of significant effects of response type across the entire epoch and displays the *p-values* as a two-dimensional statistical color-scaled map [see Statistical Cluster Plots (SCPs)].

#### Topographic Mapping

Brain Electric Source Analysis software (BESA; MEGIS Software GmbH), was used to generate topographical distributions of the multisensory summed and simultaneous conditions over predefined time windows of interest for aligned and misaligned pairs. Difference waves of the simultaneous (VS) minus summed (V + S) conditions for aligned and misaligned pairs were calculated and the integrative effects of the respective distributions were also topographically mapped.

## Results

### Behavioral Results

Participants easily detected stimuli from each modality, responding successfully to 95 ± 0.36% (mean ± SEM) of the somatosensory stimuli, 94.8 ± 0.35% of the visual stimuli, and 95.1 ± 0.37% of the multisensory stimulus pairs. We conducted an ANOVA to test for differences in RTs based on stimulus condition (unisensory V, unisensory S, aligned VS, or misaligned VS multisensory pairs) and stimulus presentation side (left or right hemifield). Results indicated a main effect of stimulus type (*F*_3,11_ = 124.22, *p* < 0.01). No other main effects or interactions were found. We then conducted a second ANOVA to test for significant differences in RT between spatially aligned and spatially misaligned VS stimulus pairs. The within-subject factors included type of VS stimulation (aligned or misaligned) and hemifield of somatosensory stimulation (left or right). Results indicated no significant difference between aligned and misaligned stimulus conditions (*p* = 0.14); suggesting that the mean RTs for all four VS stimulus pairs were not significantly different from each other.

In order to assess the reliability of the redundant sensory effects (RSEs), eight separate *t*-tests (i.e., planned comparisons) between each of the unisensory stimulus conditions and the simultaneous multisensory stimulus conditions were performed. Results revealed that mean RT for each multisensory condition was significantly faster than the mean RTs of the constituent unisensory stimuli (see **Table 1**), and these findings remain even after application of a Bonferroni correction (*p* < 0.006). These results suggest that regardless of the side of space that the stimuli were presented, a facilitation of RT for multisensory stimuli *vs.* unisensory stimuli was present.

**FIGURE 2:**
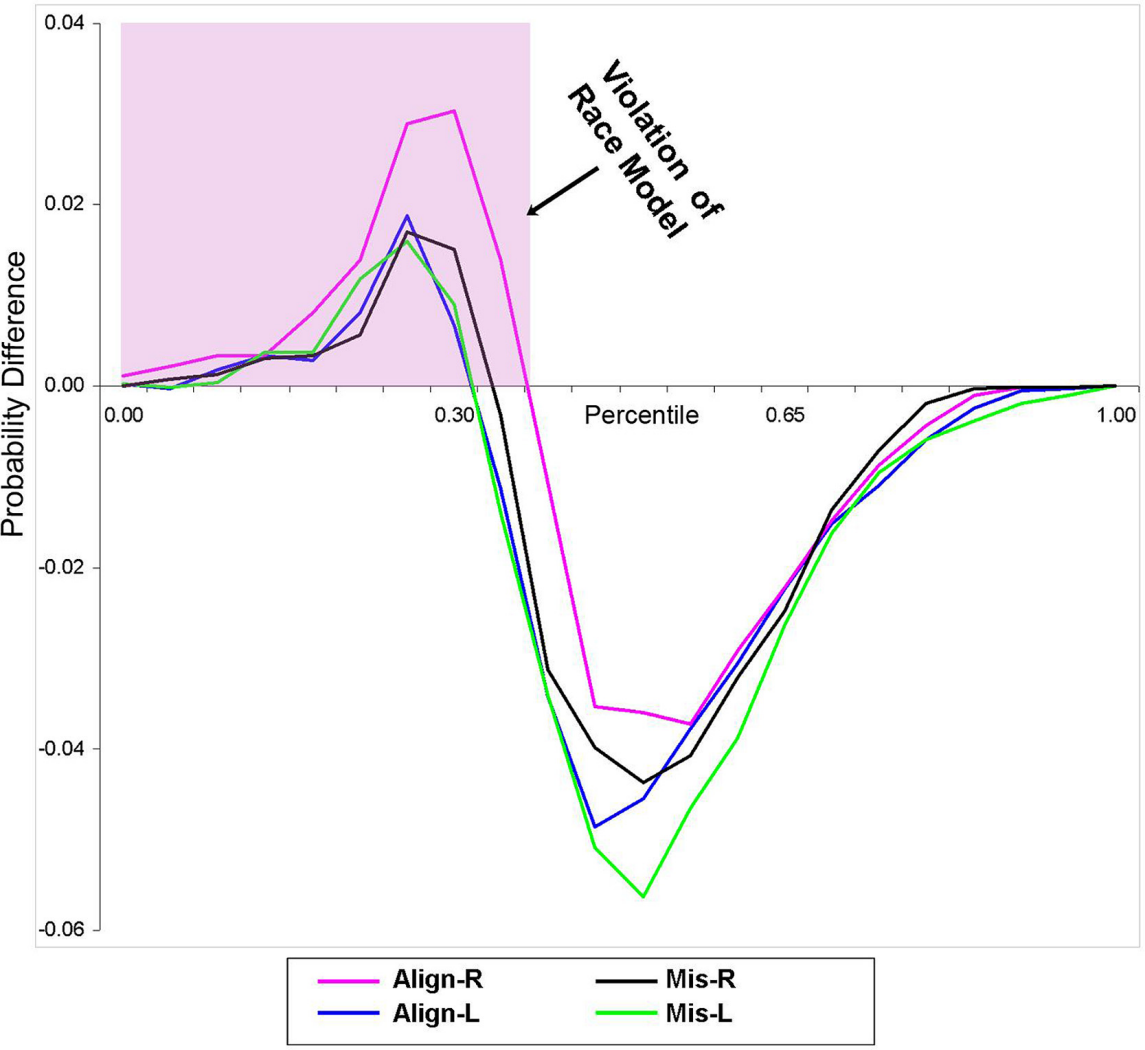
**Test of the race model.** Difference waves between actual values of multisensory VS conditions vs. the predicted values using Miller’s (1982) inequality are plotted. Any value greater than zero indicates a violation of the race model. The pink highlighted box depicts a violation of the race model in support for coactivation, and this violation was obtained in all four multisensory experimental conditions.

Using Miller’s (1982) inequality, we tested whether the RSE exceeded the statistical facilitation predicted by probability summation. The CP at each quantile was group-averaged separately for each stimulus condition to form a distribution that maintained the shape of the individuals’ data and was then compared to the model. Behavioral results from this study indicate that the race model was indeed violated (i.e., values greater than zero) in all four conditions over the first 30% of the grouped (*n* = 14) RT distribution (**Figure 2**).

### Unisensory Electrophysiological Results

**FIGURE 3:**
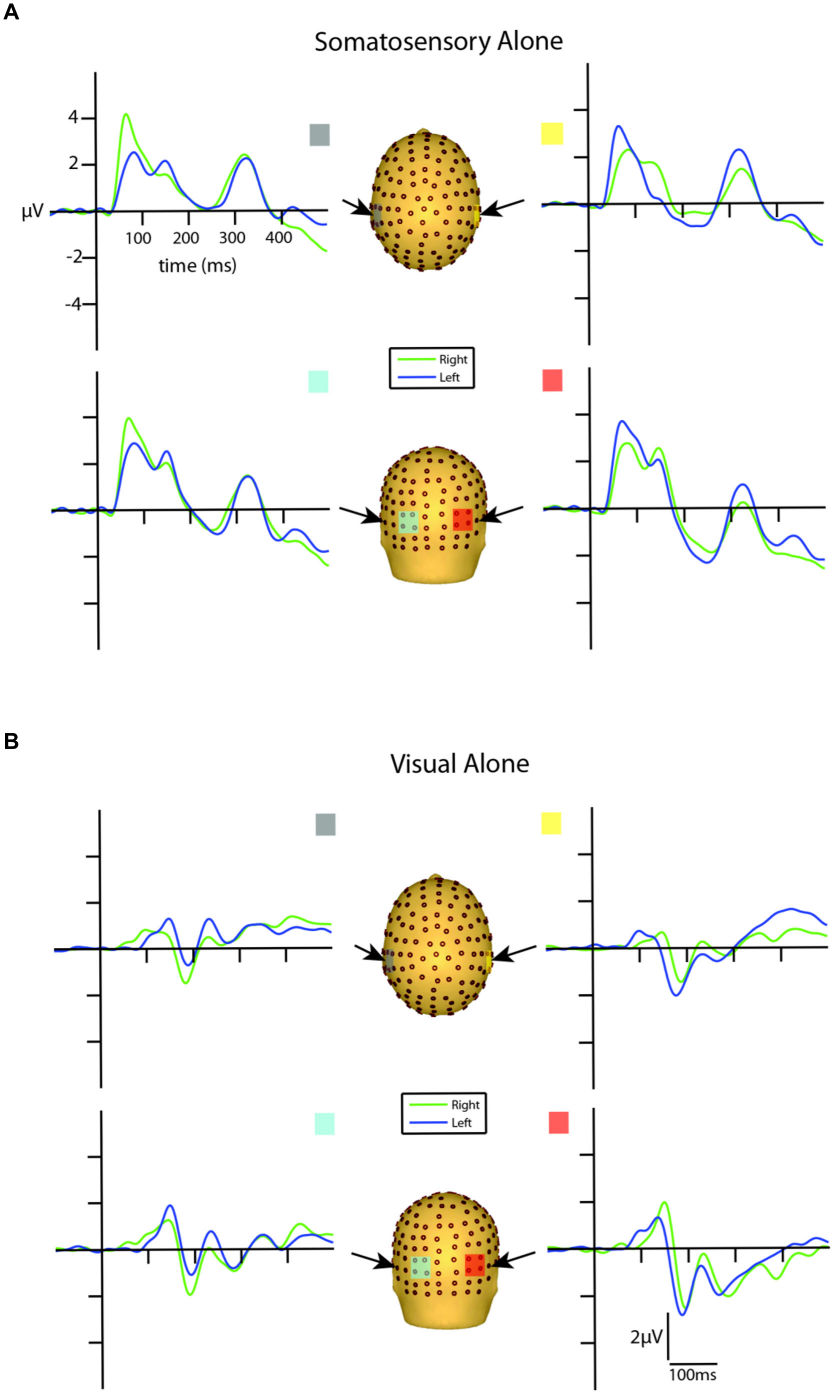
**Unisensory ERPs for selected ROIs. (A)** Unisensory somatosensory ERPs for stimuli presented to the right (green traces) and left (blue traces) hemifield. **(B)** Unisensory visual ERPs for stimuli presented to the right (green traces) and left (blue traces) hemifield. All axes are the same and the labels are provided in the top left corner.

Visual inspection of the unisensory somatosensory evoked potentials (SEPs) revealed a robust P60 component emerging at around 30 ms and reaching its peak at ∼65 ms, followed by an N140 component peaking at ∼135 ms over lateral central and posterior scalp sites on the hemisphere contralateral to the stimulated hand (see **Figure 3A**). These latencies are consistent with other studies that employ vibro-tactile stimuli (see Tobimatsu et al., 1999).

Inspection of the VEPs elicited during the visual alone conditions revealed a P1 component emerging from baseline at around 80 ms, reaching its peak at about 135 ms, with maximal distribution over occipital scalp. The P1 was followed by an N1 that reached its peak at ∼185 ms (see **Figure 3B**). The late onset of these components is a reflection of the physical properties of the visual stimuli employed in the current study (i.e., small LEDs presented quite peripherally at 25° from central fixation), consistent with Busch et al. (2004) who showed that as the eccentricity of two visual stimuli increased from central to peripheral locations, P1 amplitude decreased and latency increased.

In order to determine the earliest detectable onset of the VEP, a point-wise running *t*-test analysis (two-tailed) was implemented to calculate the statistical differences of the unisensory visual left and right conditions from the zero baseline across all 14 participants. Onset time was defined as the first point of 10 consecutive data points (i.e., 10 data points = 19.53 ms, at a digitization rate of 512 Hz) meeting an alpha criterion of 0.05. The first detectable response to the left visual alone condition onset at 87 ms over both contralateral and ipsilateral scalp sites. The onset of the first detectable visual response to right-sided stimulation was observed somewhat earlier at 58 ms over both contralateral and ipsilateral scalp sites. After corroborating these findings by inspecting the morphology of the group-averaged waveforms, we determined that an additional examination for putative multisensory effects during this initial visual response window (80–100 ms) was merited. This period is consistent with the timeframe of the visual C1 component of the VEP (Kelly et al., 2008).

### Multisensory Visual-Somatosensory Interactions

**FIGURE 4:**
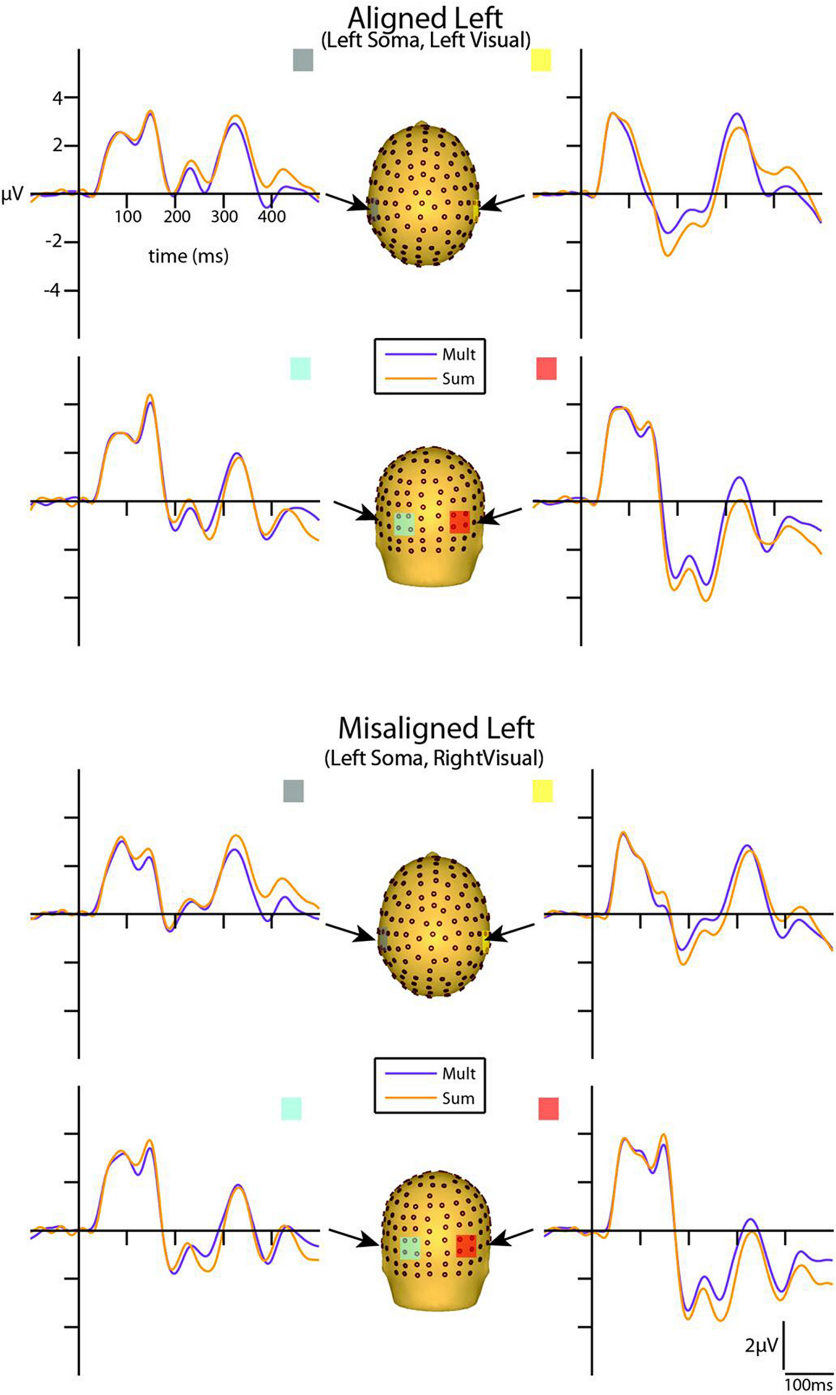
**Simultaneous vs. summed MSI effects – left conditions for selected ROIs.** ERP waveforms of both the aligned left and misaligned left visual-somatosensory (VS) conditions are depicted for the four ROI. Purple traces represent multisensory simultaneous VS activity and orange traces represent summed V + S activity. All axes are the same and the labels are provided in the top left corner.

**FIGURE 5:**
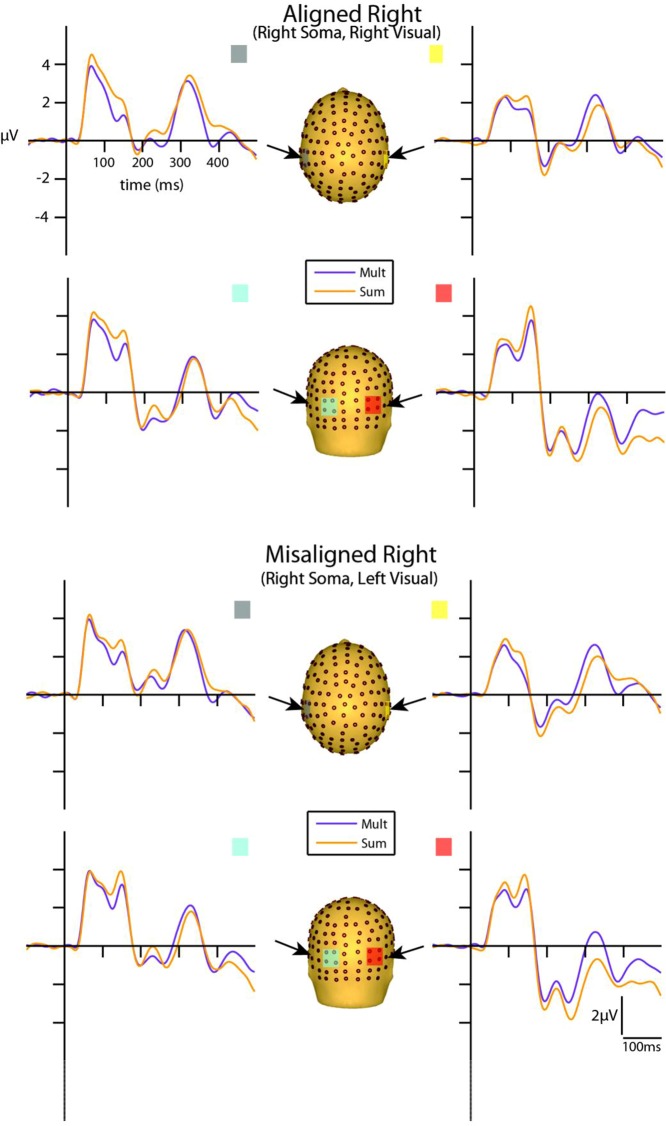
**Simultaneous vs. summed MSI effects – right conditions for selected ROIs.** ERP waveforms of both the aligned right and misaligned right VS conditions are depicted for the four ROI. Purple traces represent multisensory simultaneous VS activity and orange traces represent summed V + S activity. All axes are the same and the labels are provided in the top left corner.

Visual inspection of the group-averaged ERPs for simultaneous VS and summed V + S aligned conditions revealed differences in amplitude starting at around 55 ms for the right conditions and 85 ms for the left conditions over respective contralateral hemispheres (recall that side of presentation in misaligned conditions is with reference to the side of somatosensory presentation; see **Figures 4** and **5**). The first differences between simultaneous and summed neural activity appeared maximal over contralateral left central-parietal regions for the aligned right condition. More specifically, the ERP elicited to the V + S right condition was more positive in amplitude than the ERPs elicited to the simultaneous VS right condition over contralateral scalp regions. These initial divergences between 55 and 75 ms were not apparent in the aligned left or the misaligned conditions.

However, divergences between the simultaneous and summed multisensory conditions appeared most prominent during a time window of 110–130 ms, over both central-parietal and occipital scalp regions. Such robust differences between simultaneous and summed neural activity were apparent for both left and right conditions, regardless of spatial alignment. During this time interval, the summed conditions were consistently of greater amplitude than the simultaneous conditions. **Figure 4** displays simultaneous (purple traces) *vs.* summed (orange traces) multisensory ERPs over the specified ROIs for the spatially aligned and misaligned left pairs. Similarly, **Figure 5** depicts simultaneous *vs.* summed multisensory ERPs over the specified ROIs for the spatially aligned and misaligned right pairs.

As detailed above, the following five 20 ms time windows (i.e., time windows around various unisensory components) were pre-selected for testing over appropriate scalp regions: (1) the somatosensory P60 (55–75 ms), (2) the somatosensory N1 (125–145 ms), (3) the visual C1 (80–100 ms), (4) the visual P1 (125–145 ms) and (5) the visual N1 (180–200 ms). In addition, one *post hoc* exploratory time window was also selected for testing over central-parietal scalp regions during a time window of (110–130 ms), since clear divergences during waveform inspection were noted between simultaneous and summed multisensory pairs and warranted statistical analysis. **Table 2** delineates the statistical results for the various ANOVAs conducted on the multisensory and summed electrophysiological data over these six time windows.

**Table 2:** Electrophysiological results^**b**^.

ERP component	ROI	Latency (ms)	Factors				Interactions
			Condition	Alignment	Stimulus Side	Hemi-scalp (ROI)	
**Central Positivity** Somatosensory P60	Central-Parietal	55–75	NS	NS	NS	***F*(1,13) = 8.13, *p* ≤ 0.05**	Condition × Stimulus side ***F*(1,13) = 5.42, *p* ≤ 0.05** Stimulus Side × ROI ***F*(1,13) = 94.68, *p* ≤ 0.01**
**Central Integrative** **Negativity**	Central-Parietal	110–130	***F*(1,13) = 10.87, *p* ≤ 0.01**	NS	NS	NS	Condition × Stimulus Side × ROI ***F*(1,13) = 12.55, *p* ≤ 0.01**
**Central Negativity** Somatosensory N140	Central-Parietal	125–145	***F*(1,13) = 17.52, *p* ≤ 0.01**	NS	NS	NS	Condition × Stimulus Side ***F*(1,13) = 10.89**, *p* ≤ 0.01 Stimulus Side × ROI ***F*(1,13) = 8.44, *p* ≤ 0.05**
**Parieto-Occipital Positivity** Visual C1	Occipital	80–100	NS	NS	NS	NS	Stim Side × ROI ***F*(1,13) = 21.64, *p* ≤ 0.01** Alignment × Stimulus Side × ROI ***F*(1,13) = 22.23, *p* ≤ 0.05**
**Parieto-Occipital Positivity** Visual P1	Occipital	125–145	***F*(1,13) = 17.52, *p* ≤ 0.01**	NS	NS	NS	Condition × Stimulus Side ***F*(1,13) = 10.98, *p* ≤ 0.01** Stim Side × ROI ***F*(1,13) = 8.45, *p* ≤ 0.05**
**Parieto-Occipital Negativity** Visual N1	Occipital	180–200	NS	NS	NS	***F*(1,13) = 26.62, *p* ≤ 0.01**	Condition × ROI ***F*(1,13) = 14.25, *p* ≤ 0.01** Alignment × Stimulus Side × ROI ***F*(1,13) = 5.84, *p* ≤ 0.05**

*^b^ Depicts the various repeated-measure ANOVAs results for the central-parietal and occipital regions of interest (ROI) over six pre-defined latencies.*

#### Central-Parietal Visual-Somatosensory Interactions

Differences between simultaneous and summed multisensory conditions over contralateral and ipsilateral central-parietal scalp regions around the somatosensory P60 (55–75 ms) were investigated. Results revealed a main effect of ROI (*F*_1,13_ = 8.13, *p* ≤ 0.05). The interaction of condition x stimulus presentation side was significant (*F*_1,13_ = 5.42, *p* ≤ 0.05) and suggested greater multisensory integrative effects for the right as compared to left stimulus presentation sides (see also **Figures 4** and **5**). Four follow-up ANOVAs, one for each multisensory condition (i.e., aligned right, aligned left, misaligned right, and misaligned left) with factors of condition (multisensory or summed) and ROI (left or right hemi-scalp) were conducted to further understand the basis for this interaction effect. Results revealed a main effect of condition for only the aligned right condition (*F*_1,13_ = 7.12, *p* ≤ 0.05). In the case of the aligned left (*F*_1,13_ = 0.20, *p* = 0.66), the misaligned right (*F*_1,13_ = 0.86, *p* = 0.37), and the misaligned left conditions (*F*_1,13_ = 0.65, *p* = 0.43), this early effect was not observed. A stimulus presentation side × ROI interaction was also significant (*F*_1,13_ = 94.68, *p* ≤ 0.01) and was not surprising given that cortical activity in response to the somatosensory stimulation was greater over the contralateral hemi-scalp.

Examination of the somatosensory N140 component during the latency of 125–145 ms over central scalp regions revealed a main effect of condition (*F*_1,13_ = 10.87, *p* ≤ 0.01), with no significant effect of stimulus alignment. This result confirmed the presence of a multisensory effect over central-parietal scalp regions during this time window that was of significantly less amplitude than the summed neural response across all four multisensory conditions, regardless of spatial alignment. Similar to the P60, the somatosensory N140 also revealed significant interaction effects of condition × stimulus side, and stimulus presentation side × ROI (see **Table 2**).

#### Occipital Visual-Somatosensory Interactions

Differences between simultaneous and summed multisensory conditions centered on the first detectable visual response (i.e., the C1 component) were investigated during the time window of 80–100 ms over occipital scalp regions. Results revealed no significant main effect or interactions with condition, indicating no evidence for multisensory integrative effects over occipital cortex during the earliest detectable activation of the VEP. However, the interactions of stimulus presentation side × ROI and alignment × stimulus presentation side × ROI were significant (see **Table 2**).

Differences between simultaneous and summed multisensory conditions centered on the visual P1 component, during the time window of 125–145 ms over occipital scalp regions, were subsequently investigated. Results revealed a main effect of condition (*F*_1,13_ = 17.52, *p* ≤ 0.01). This was due to the presence of a multisensory effect over occipital brain regions during this time window, with simultaneous presentations resulting in significantly lower amplitudes than the summed neural response across all four multisensory conditions, regardless of spatial alignment. However, there were also significant interactions of condition × stimulus side (*F*_1,13_ = 10.98, *p* ≤ 0.01), driven by the fact that there were greater differences in multisensory effects over contralateral scalp regions for stimulus conditions containing right-sided somatosensory stimulation as compared to those containing left-sided somatosensory stimulation. A stimulus presentation side × ROI (*F*_1,13_ = 8.45, *p* ≤ 0.05) interaction was also significant, consistent with the observation that cortical activity in response to somatosensory stimulation was greater over contralateral hemispheres.

Examination of differences between simultaneous and summed multisensory conditions around the visual N1 component over occipital scalp regions during the latency of 180–200 ms revealed a main effect of ROI (*F*_1,13_ = 26.62, *p* ≤ 0.01). In addition, the interactions of condition × ROI (*F*_1,13_ = 14.25, *p* ≤ 0.01) and alignment × stimulus presentation side × ROI (*F*_1,13_ = 5.84, *p* ≤ 0.05) were also significant (see **Table 2**).

#### Exploratory Analysis of Visual-Somatosensory Interactions

Lastly, we evaluated differences in VS multisensory activation between simultaneous and summed multisensory pairs over central scalp regions during a time window of 110–130 ms since *post hoc* inspection of the group-averaged data suggested that this was a period of particularly robust multisensory interactions. Results revealed a main effect of condition (*F*_1,13_ = 10.87, *p* ≤ 0.01). In addition, a condition × stimulus presentation side × ROI interaction (*F*_1,13_ = 12.55, *p* ≤ 0.01) was also significant and suggested greater differences in multisensory effects over contralateral brain regions for stimulus conditions containing right-sided somatosensory stimulation as compared to those containing left-sided somatosensory stimulation. Similar to the multisensory effects for the somatosensory N140 and the visual P1, the main effect of condition during this integrative time window revealed a significant difference in multisensory compared to sum visual and somatosensory neural activation over centro-parietal regions. The condition × stimulus presentation side interaction was also significant (see **Table 2**). Again, the neural activity to the simultaneous VS stimulation was of significantly lower amplitude than the neural activity to summed V + S stimulation across all multisensory conditions, regardless of spatial alignment.

#### Statistical Cluster Plots (SCPs)

**FIGURE 6:**
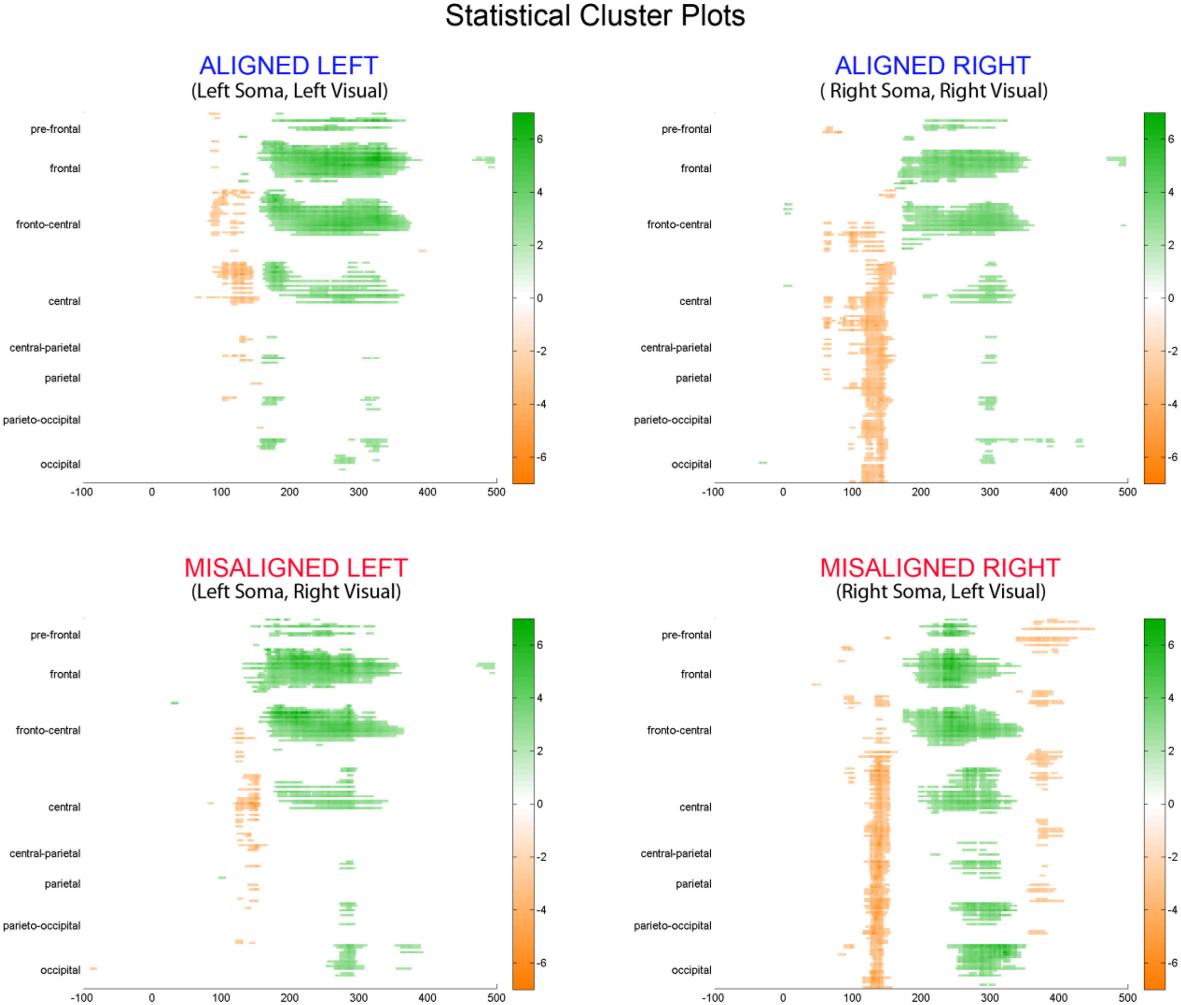
**Statistical cluster plots (SCPs).** Differences between simultaneous and summed VS activity are depicted for the aligned and misaligned conditions. Color values indicate significant *t*-values at the *p* ≤ 0.05 that resulted from point-wise running *t*-tests across time (*x*-axis) and electrode positions (*y*-axis). Electrode positions are arranged from pre-frontal to occipital regions (top to bottom *y*-axis). Within each general region, electrode laterality is arranged from right (R) to left scalp (L).

**FIGURE 7:**
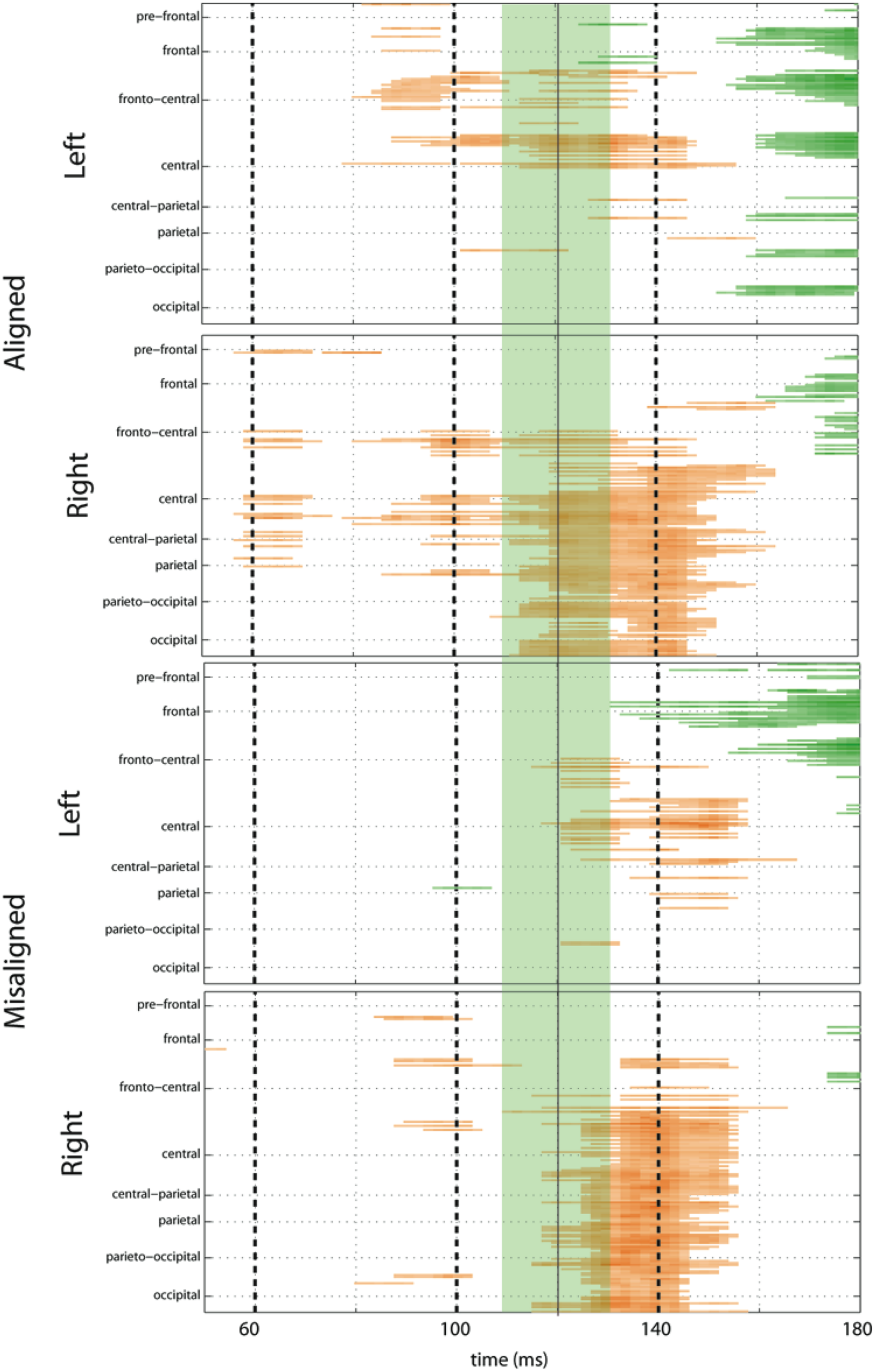
**Amplified SCPs.** Differences between simultaneous and summed VS activity are depicted for aligned and misaligned conditions during a time window of 50–180 ms. This figure illustrates three major points: (1) consistent interactions of visual and somatosensory processes across all four multisensory conditions starting at around 110 ms (green shaded area), regardless of spatial alignment; (2) that integrative effects were clearly stronger for the aligned as compared to misaligned conditions; and (3) integrative effects were noticeably stronger in the two conditions where somatosensory information was presented to the right hemi field.

To fully explore the spatiotemporal characteristics of the multisensory response, SCPs representing significant results of running *t*-tests between the simultaneous VS and the summed V + S activity were generated for each of the four multisensory conditions across all time points (between 100 ms pre-stimulus onset and 500 ms post-stimulus onset) and the entire electrode array (**Figure 6**). **Figure 7** represents an enlargement of the SCP plots focused around the earliest time period (50–180 ms) where neural differences between summed and simultaneous VS conditions were noted. This analysis revealed consistent interactions of visual and somatosensory processes across all four multisensory conditions starting at around 110 ms over central and central-parietal areas. Note that the 120 ms time point is also the midpoint of the exploratory 110–130 ms time window that was identified *post hoc* as a window of particularly vigorous integrative processing during waveform inspections (highlighted in green in **Figure 7**). Such integrative effects persisted until about 150 ms over central, central-parietal, and parietal regions for all four multisensory conditions.

The SCPs also serve to emphasize some important differences between the four multisensory conditions. Particularly noteworthy was the fact that integrative effects were clearly more robust for the aligned compared to misaligned conditions; especially the aligned right condition, where differences between simultaneous VS and summed V + S activity occurred as early as 55 ms. This multisensory effect is consistent with the results of the somatosensory P60 ANOVA reported above. Furthermore, both aligned conditions revealed integrative VS activity starting around 85 ms over contralateral hemi-scalp that was not as clearly seen in the case of both misaligned conditions. This relatively weak effect was distributed across a small number of channels and *post hoc* analyses during the 85–105 ms time window did not reveal significant effects of alignment (*F*_1,13_ = 1.52, *p* = 0.24) over our pre-defined central ROIs. Nonetheless, clear multisensory effects over central-parietal regions during the 110–150 ms time window were significant regardless of spatial alignment; however, these effects were noticeably more robust in the two conditions where somatosensory information was presented to the right hand. This finding suggests that handedness may have played a role in multisensory processes involving somatosensory stimulation; note all participants were right handed. Similarly, inspection of the later integrative effects centered around the visual P1 revealed that conditions containing right somatosensory presentations manifested robust integrative effects that were widespread across multiple channels over visual regions. However, the conditions containing left hemispheric somatosensory presentations evidenced no significant integrative effects over these parietal and occipital brain regions (see **Figures 6** and **7**).

### Topographic Mapping Results

**FIGURE 8:**
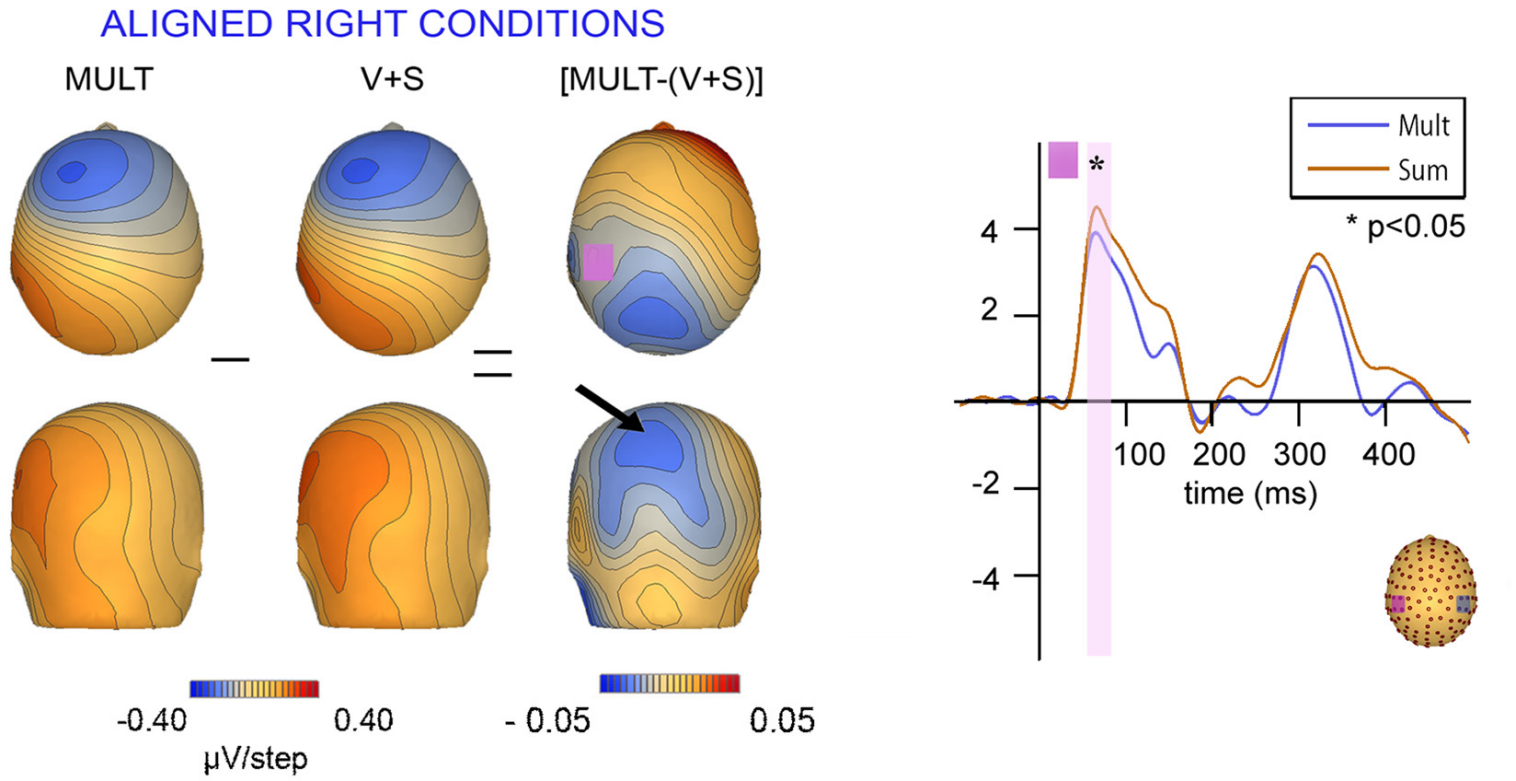
**Scalp topographies at 65 ms and corresponding P60 waveform.** Scalp topographies during the time window of the P60 (55–75 ms) for simultaneous and summed VS conditions for the Aligned Right Conditions over the left centro-parietal ROI. The pink highlighted bar of the waveform depicts significant difference in neural activation between multisensory *vs.* summed conditions over the 55–75 ms time period.

Scalp topographies of VS integrative processing effects for the early Aligned Right MSI effect (time window 55–75 ms) were mapped in BESA and are depicted in **Figure 8**. Inspection of the scalp topographies during this time frame revealed robust lateralized VS integration activity for simultaneous and summed VS conditions over the contralateral central-parietal scalp (i.e., left hemi-scalp) to the stimulated hand. This activity was stronger for the V + S condition and resulted in a weaker bifocal negative complex evident over central parietal and left temporal scalp regions (**Figure 8**, black arrow). The associated EEG activity, right panel of **Figure 8** (highlighted in pink), depicts the significant multisensory integrative effects reported in the above referenced ANOVAs.

**FIGURE 9:**
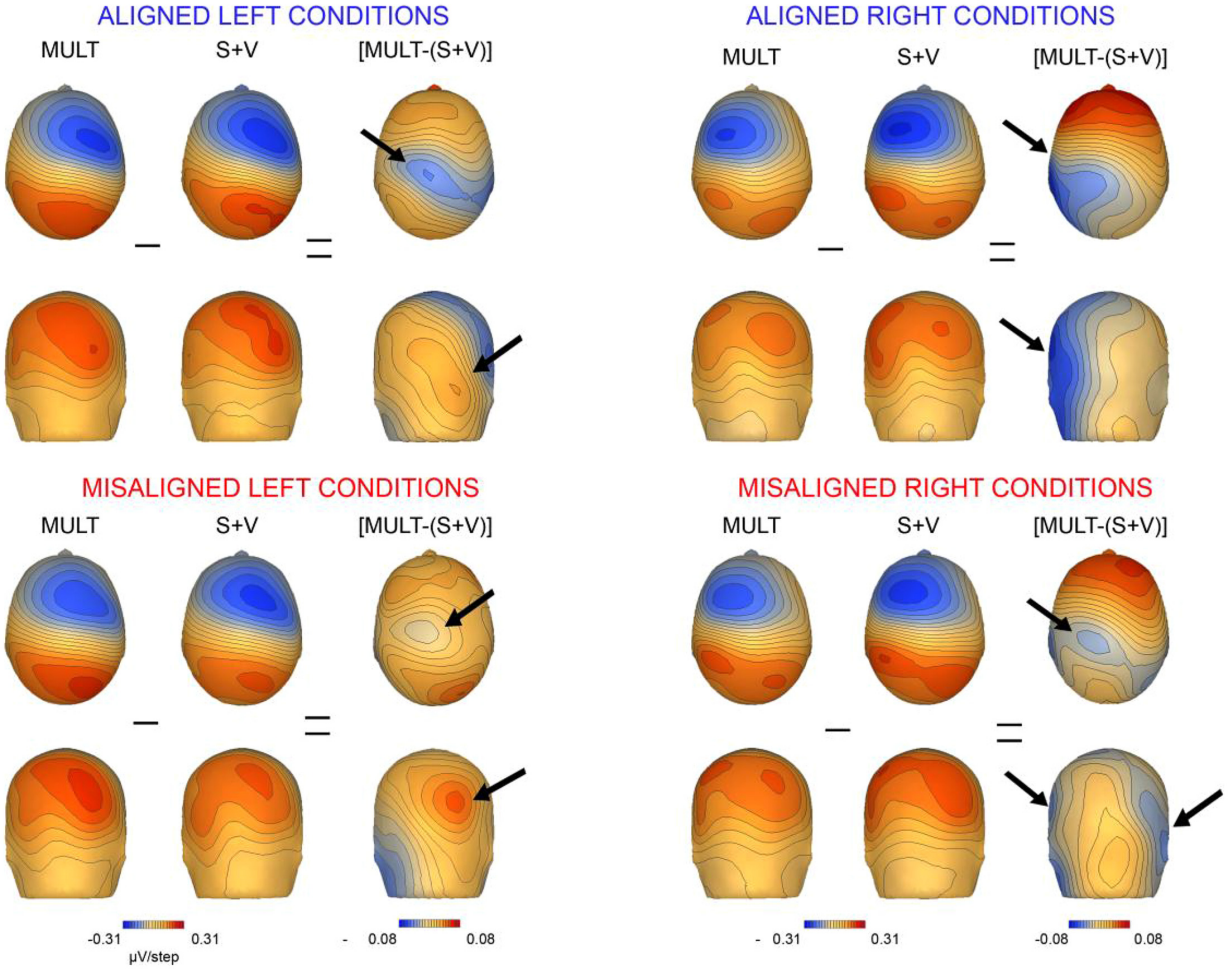
**Scalp topographies.** Scalp topographies at 120 ms during the VS integration time window of (110–130 ms) for simultaneous and summed VS conditions. The aligned conditions are presented on the top and the misaligned conditions are presented on the bottom. The arrows depict the bifocal negative complex that was present for each VS condition.

We also mapped the scalp topographies of VS integrative processing effects that were common across all four multisensory conditions (i.e., those seen in the 110–130 ms timeframe). Inspection of the scalp topographies at 120 ms during the exploratory VS integration interval for simultaneous and summed VS conditions revealed lateralized activation over the contralateral central-parietal scalp to the stimulated hand for both left and right conditions, regardless of spatial alignment (see **Figure 9**). Noteworthy is the similar activation over contralateral right hemi-scalp for aligned left and misaligned left conditions, with almost mirror image distributions over contralateral left hemi-scalp for aligned right and misaligned right conditions.

Overall, inspection of the VS integration effects during this latency revealed similar VS integration effects across spatially aligned and misaligned pairs, but some differences were also noted. First, within the aligned conditions, a large bifocal negativity was evident over contralateral central scalp, extending down over the temporal scalp, and this net negativity switched between hemispheres in an essentially symmetrical fashion, in keeping with the hemifield of stimulation (**Figure 9**). For the misaligned conditions, a similarly distributed but weaker bifocal negative complex was also evident over central and lateral temporal scalp regions, which occurred on the contralateral hemisphere of the stimulated hand. The VS integration effect ([Mult- (S + V)]) over centro-parietal regions was similar across all four conditions; however, these effects were most robust for the aligned right condition where no sensory information was presented to the left hemifield (**Figure 9**). Collectively, the appearance of VS integration effects across all four conditions reveals that spatial alignment of VS information is not critical for MSI during the 110–130 time intervals.

## Discussion

The aims of the present study were two-fold: our first goal was to determine the spatial and temporal properties of cortical VS multisensory interactions in humans, while our second goal was to determine whether the earliest stages of VS integrative processing in cortex would also occur if visual and somatosensory stimuli were spatially misaligned. Both behavioral and electrophysiological results from the current study provide evidence for extensive VS interactions regardless of whether the constituent inputs were aligned or not. At the behavioral level, participants were significantly faster at responding to VS multisensory conditions than to either of the constituent unisensory conditions, again regardless of spatial location. These speeded responses to all four VS conditions, indicative of the so-called RSE, also violated the race model. That is, these violations confirmed that the RSE could not be accounted for by simple probability summation, consistent with previous work using other sensory pairings (Molholm et al., 2002; Murray et al., 2005; Brandwein et al., 2011, 2013; Girard et al., 2011; Mahoney et al., 2011; Megevand et al., 2013; Andrade et al., 2014). Thus, the current behavioral results suggest that visual and somatosensory neural responses interacted to produce significant RT facilitation, regardless of spatial alignment.

Turning to the electrophysiological results, a considerably more nuanced picture emerged. While several phases of MSI were observed for all four spatial combinations, there were also observable differences in the timing and robustness of these effects. First, the earliest multisensory effect was detected at just 55 ms, but this was only the case when the somatosensory and visual elements were presented in the right hemifield (i.e., in the aligned-right condition). A second wave of relatively weak multisensory effects (85–105 ms), over central and fronto-central regions, was uncovered during *post hoc* analyses. These effects were seen contralateral to the stimulated hand and appeared to be more extensive and robust for the aligned conditions. However, additional *post hoc* analyses failed to establish an effect of alignment, so these differences must be interpreted with caution. In turn, a period of robust integrative processing between ∼110–150 ms was evident for all four spatial combinations. Interestingly, this effect was substantially more robust in the two conditions where somatosensory inputs were presented to the right hand, rather than the two conditions where inputs were aligned. As such, side of presentation appears to have been as important a factor in driving visuo-somatosensory interactions as spatial alignment during this early period. These results are in keeping with findings reported by Macaluso et al. (2000) and Macaluso and Driver (2001) where multisensory visuo–tactile responses were localized to higher association areas (i.e., the anterior part of the intraparietal sulcus) in studies examining crossmodal links during voluntary endogenous attention. In what follows, we unpack these effects as well as later processes in more detail and relate them to findings from the extant literature.

### “Early” Visuo-Somatosensory Integrative Effects

As mentioned, the earliest detectable multisensory effect occurred at 55 ms over contralateral left centro-parietal regions, solely for the aligned-right condition. While this result would appear to point to alignment as a major organizing principle in early VS integration, the fact that the effect was not observed for the aligned-left condition suggests otherwise. Our suspicion is that this effect likely stems from the fact that all participants in this study were right-handed. Prior work has shown that right-handers have greater cortical somatosensory representations compared to left-handers (Buchner et al., 1995; Soros et al., 1999), which might explain why a relatively weak effect such as this was only observed for the aligned-right condition. Work from Sieben et al. (2013) has shown that visual stimulation affects tactile processing by modulating already active network oscillations in S1 via cortico-cortical and subcortical feedforward interactions, providing a plausible neural substrate for these effects. Note that we have observed similar cross-sensory oscillatory coupling between the auditory and visual systems using direct intracranial electrocorticographic recordings in human epilepsy patients (Mercier et al., 2013). However, another plausible explanation for this unilateral effect may relate to the well-established hemispheric asymmetries in spatial attentional processes, although these typically lead to a left visual field bias in right-handers (e.g., Foxe et al., 2003; Marzoli et al., 2014), whereas the current results would seem to point to a right field advantage for MSI.

Our main analyses were initially restricted to a limited set of time periods and scalp regions based on the well-characterized unisensory components of the visual and somatosensory ERPs. This was mainly because of the very limited existing ERP literature investigating VS integrations. That is, we had very little to go on in deriving our initial hypotheses. Consequently, we also employed the SCP technique to explore the entire data matrix for other periods of potential multisensory integrative processing. Given the paucity of previous work, this was clearly warranted, since overly restrictive analyses would be almost certain to result in Type II errors. Of course, it bears re-emphasizing that any and all effects uncovered through this technique must be considered *post hoc* and will bear replication in further studies before any strong conclusions can be justified. In this spirit, SCP analysis revealed a second phase of relatively weak multisensory interactions beginning at 85 ms in a cluster of channels over central-parietal regions for the aligned conditions. Similar effects were not evident for the misaligned stimulus conditions. A main effect of condition was confirmed by ANOVA, but the interaction of alignment by condition did not reach significance. Thus, while the SCPs suggest that alignment plays a role in the interaction effects seen during this early period, this could not be confirmed, and it will fall to future work to both confirm the effects seen during this period and to more fully interrogate the role of alignment.

In turn, significant differences between simultaneous and summed neural activity were observed in the period between 110 and 130 ms over central-parietal regions regardless of spatial alignment. Inspection of data during this time window was driven primarily by the SCPs where group-averaged data revealed robust multisensory interactions that warranted further analysis. While multisensory effects were significant for all four spatial combinations, the more robust integrative VS effects were evident for the conditions containing right somatosensory presentations. Further the most robust integration effect over contralateral central cortex during this time window was seen in the aligned-right condition.

It is of interest to compare the timing of multisensory effects reported here to those found for other sensory pairings. Robust integration effects have been seen at ∼50 ms for auditory-somatosensory pairings (Foxe et al., 2000; Murray et al., 2005), and also at 50 ms for audio-visual pairings (Molholm et al., 2002), whereas the emergence of robust effects that could be observed in all four conditions was not until after 100 ms in the current study. We believe that this is directly related to the physical properties of the visual stimuli employed in the current study. That is, small LEDs were placed directly above the somatosensory vibrators, which were both mounted to the participants’ hands to ensure that the unisensory stimuli were delivered to the same exact spatial location. Participants were required to fixate a cross on the computer monitor and not look directly at the LEDs, which were presented 25° from central fixation. The use of a minimally effective visual input at a peripheral location where visual sensitivity is relatively poor, is a possible reason why the onset of the earliest detectable visual responses was late relative to previous work (i.e., 58 ms for right hemifield stimulation and 87 ms for the left hemifield; see e.g., Frey et al., 2013). Unisensory visual and somatosensory activation to stimuli presented to the right hemifield onsets before 60 ms, thus affording the possibility of VS integration during the time window of the somatosensory P60. However, multisensory VS processing cannot be expected to occur (or at least to be detectable) before there is a detectable visual response in cortex, as was the case for the earliest observable response to left visual presentations (87 ms). Thus, it is perhaps not surprising that complementary multisensory effects were not found in the aligned left conditions in the time window from 55 to 75 ms.

### “Later” Visuo-Somatosensory Integrative Effects

Additional time windows centered on the somatosensory N140, visual P1, and visual N1 components were also tested to determine the presence or absence of multisensory integrative processing. Results revealed significant differences between simultaneous and summed neural activity during the latency range of 125–145 ms for the N140 and P1 components, where greater multisensory integrative effects were observed for conditions containing somatosensory stimulation to the right hemifield. No integration effects were found during the 180–200 ms time window of the visual N1.

Inspection of the SCPs during the time interval of the somatosensory N140 for simultaneous and summed VS conditions revealed significant multisensory integrative effects for all four experimental conditions over central-parietal regions. However, the conditions containing right somatosensory presentations demonstrated robust integrative effects that were widespread across multiple channels. Conversely, the conditions containing left hemispheric somatosensory presentations maintained more focal integrative effects around central scalp regions that were of less intensity.

In terms of the visual P1, the SCPs revealed a somewhat similar finding where the conditions containing right somatosensory presentations demonstrated robust integrative effects that were widespread across multiple channels over contralateral parieto-occipital and occipital regions. However, this was simply not the case for the conditions containing left hemispheric somatosensory presentations, as evidenced by the lack of significant integrative effects across channels in the SCPs (see **Figures 6** and **7**). In this case, the main effect of the multisensory condition is likely explained by the interaction of condition × stimulus presentation side during this 125–145 time interval over visual areas.

The finding of a prominent focus over contralateral parieto-occipital scalp is consistent with the notion of a generator in the vicinity of the inferior parietal lobe, although any inferences about intracranial sources made on the basis of topography must be treated with a large degree of caution. Nonetheless, this finding is certainly consistent with findings of VS integration in the intraparietal sulcus of the rhesus monkey as demonstrated by Hyvärinen and Shelepin (1979) and Seltzer and Pandya (1980). However, this effect requires replication as we did not make any specific hypotheses concerning it, except insofar as a complex system of integrations was expected based on our previous observations (Molholm et al., 2002).

### Limitations and Future Directions

The purpose of the current study was to determine the spatial and temporal properties of VS integration in young adults and to assess whether spatial alignment is critical for the occurrence of early VS interactions. While results from our study reveal that spatial alignment is not critical for early VS interactions; this study is not without its limitations. Given the finding that our right-handed cohort demonstrated a unique multisensory benefit during the somatosensory P60 for spatially aligned VS information presented to the right hemifield, it would be of significant interest to determine whether a left-handed cohort would demonstrate a unique multisensory benefit during this time interval when processing spatially aligned left-sided VS information. Additionally, a larger sample size of both right and left handers would be required to reliably determine whether the early multisensory effect at 55 ms is solely dependent upon handedness, whether spatial alignment of inputs to left-handers would result in a mirroring of this result, or if there is potentially a right hemifield advantage for MSI of visuo-tactile inputs.

It is also worth noting that although we refer to multisensory inputs that occur on the same side of space as spatially aligned in the current study, the experimental setup did not allow for stimuli to be presented to precisely the same spatial location due to the size of the LED, the size of the vibrator, and the necessary use of noise-attenuating gloves (which were also necessary to preclude any possibility of visualization of the vibrations). On average, the light and vibratory inputs were ∼2.5 cm from each other, although they were certainly “aligned” in space, in that on a projection from the centrally fixating observer’s point of view, the two inputs fell along the same line. Nonetheless, we cannot rule out that even closer spatial correspondence might have further enhanced measures of integration.

Another design feature here that likely militated against our ability to detect even earlier MSI effects derives from the use of highly peripheral and relatively weak visual inputs. More peripheral inputs are represented by considerably fewer neurons in the visual cortex (Adams and Horton, 2003; Frey et al., 2013) and these representations are buried deep within the medial wall of the posterior occipital cortex, along the calcarine fissure (Wong and Sharpe, 1999), where projection to the scalp surface will be greatly attenuated. Perhaps if stimulus presentations were more central (e.g., 6–10° from central fixation) and more robust visual inputs were employed, earlier components of the VEP (i.e., the C1 component; Foxe et al., 2008) would have been evoked, allowing for better detectability of early VS interactions.

Lastly, it has been known since the early days of multisensory research that integration effects are particularly strong under circumstances where the constituent unisensory stimuli are minimally effective in evoking responses – so-called “*inverse effectiveness*” (Meredith and Stein, 1986; Stein et al., 2009; Senkowski et al., 2011); but see (Ross et al., 2007) for circumstances where this is not always the case. We did not manipulate stimulus effectiveness in the current study but it would be of significant interest to determine whether integration effects strengthen differentially for spatially aligned VS inputs relative to misaligned inputs, an obvious direction of future research.

## Conclusion

At the level of behavioral facilitation, multisensory inputs resulted in significantly speeded response times and this was the case regardless of whether the visual and somatosensory constituents of bisensory inputs were spatially aligned or misaligned, mimicking a considerable body of work using other sensory pairings (i.e., audio-visual and audio-somatosensory combinations). In turn, there were clear multisensory effects observed in the electrophysiological results for all spatial combinations of somatosensory and visual inputs. However, a degree of spatial specificity was observed in these effects during the earliest processing periods, unlike prior work using audio-somatosensory pairings. The earliest integrative effects were observed solely in the case of aligned inputs to the right hemifield (∼55 ms) and a subsequent phase of integrative processing (85–105 ms) was only observed in the case of aligned left and right sided inputs. Two somewhat later phases of integrative effects (∼110–130 ms over centro-parietal scalp and ∼125–145 ms over both central and parieto-occipital scalp) were common to both aligned and misaligned conditions, but both of these phases showed sensitivity to the hand of input, with integrative effects strongest for the two conditions where the right hand was stimulated. The current results suggest that the finer spatial tuning of the visual and somatosensory systems leads to an initial round of multisensory integrative effects that are indeed sensitive to the spatial alignment of the constituent sensory inputs, much like the effects that have been observed in animal studies in the SC (Yu et al., 2013; Xu et al., 2014). Nonetheless, considerable integrative processing was also observed for misaligned inputs, although it developed somewhat later in processing (after 100 ms). Thus, visuo-somatosensory cortical integration effects, while sensitive to spatial alignment, are not entirely constrained by the simple physical correspondence between inputs (in this case location). The data suggest that integrative processing can be evoked in the service of task completion (in this case, to respond as quickly as possible), and that such task-set configurations may allow for more flexible deployment of multisensory processing. Future work will be needed to determine if these spatially insensitive multisensory processes are observed when attention is manipulated away from the bisensory inputs (Talsma et al., 2007, 2010). The data also suggest that handedness may play a special role in visuo-somatosensory integration, since integrative processing was clearly strongest for inputs to the right hand, but this remains to be formally tested in future work.

## Author Contributions

JM, JF, and MR designed, developed, and implemented the project. JM collected the data. JF and SM supervised the project. WR provided substantial contributions to the conception and design of the project. JM, JB, and PS analyzed the data and created all figures. All authors contributed extensively to the work presented in this paper, commented on the manuscript throughout the editorial process, and approved the final submitted version.

## Conflict of Interest Statement

The authors declare that the research was conducted in the absence of any commercial or financial relationships that could be construed as a potential conflict of interest.

## Footnotes

1 Teder-Sälejärvi et al. (2002) cautioned that such early integration effects could easily be contaminated or confounded by slow anticipatory potentials that are subtracted twice in the [AV- (A + V)] difference wave. That is, slow anticipatory waves are present in the unisensory auditory, unisensory visual and multisensory AV conditions. By adding the unisensory A to the unisensory V condition, there is a doubling of anticipatory potentials that are subtracted from the multisensory AV condition, which only contains one anticipatory potential.

2 The visual stimuli were 4 cm × 4 cm square checkerboards presented on a 17-inch CRT screen. Thus, at the viewing distance used, even if these were placed at the very extreme left and right edges of the screen, they would have been approximately only 6–7° from the vertical meridian, and it seems likely that they were somewhat less.

## Abbreviations

CP: cumulative probability
ERP: event-related potentials
ITI: inter-trial-interval
LEDs: light emitting diodes
MSI: multisensory integration
RSE: redundant signals effect
SC: superior colliculus
